# The tip of the iceberg: a distinctive new spotted-wing *Megaselia* species (Diptera: Phoridae) from a tropical cloud forest survey and a new, streamlined method for *Megaselia* descriptions

**DOI:** 10.3897/BDJ.2.e4093

**Published:** 2014-11-12

**Authors:** Emily A. Hartop, Brian V. Brown

**Affiliations:** † Natural History Museum of Los Angeles County, Los Angeles, United States of America

**Keywords:** Tropical, biodiversity, taxonomy, table-based description, open-ended taxon

## Abstract

A new *Megaselia* species, *M. shadeae*, with a large, central, pigmented and bubble-like wing spot and a greatly enlarged radial wing vein fork, is described from Zurquí de Moravia, Costa Rica. As part of the Zurquí All Diptera Biodiversity Inventory (ZADBI) project, it represents the first of an incredible number of new phorid species to be described from this one Costa Rican cloud forest site. A new, streamlined method of description for species of this enormous genus of phorid flies is presented.

## Introduction

The genus *Megaselia* Rondani has been characterized as the “Diptera *enfant terrible*” (Smith 1984) and as an "open-ended taxon" (Bickel 2009). This single genus contains about half of the species of Phoridae, a majority of which are hitherto undescribed. The life histories within this single genus are more diverse than the rest of the family combined, with some *Megaselia* species being predators, parasitoids, kleptoparasites, and commercial pests. They consume fungi, plants, other insects at every stage, carrion, and many other substrates thought truly uninhabitable (Disney 1994). The ecosystem services this genus provides cannot be overestimated. Given the diversity and enormity of *Megaselia*, it is imperative that taxonomic work on this group be made a priority.

The following represents a newly discovered species with wing spots that make it an easily recognized species of *Megaselia* within the Neotropical fauna of the genus (and, indeed, even within the worldwide fauna). *Megaselia* species with wing spots are rare, and this character alone easily excludes this species from most others in the literature.

Specimens were collected from the Zurquí All Diptera Biodiversity Inventory (ZADBI) project in Costa Rica. ZADBI is an ambitious, multi-faceted study focused on generating a thorough inventory of the dipteran fauna of a specific cloud forest site using varied and complementary collection methods. The project is revealing a goldmine of new species, not least within this gargantuan genus of phorid flies. The distinctiveness and ease of identification of the species herein described, but lack of previous recognition, hints at the tremendous amount of taxonomic work needed for this group.

In work on the ZADBI Project and beyond, the authors have spent countless hours sorting through tens of thousands of worldwide *Megaselia* specimens. Recognized morphotypes are keyed and compared to published *Megaselia* descriptions in the world literature. Due to the prevalence of “tramp” species of *Megaselia* that appear and establish themselves in areas around the world, all available literature, regardless of geographic region, must be utilized to determine the status of a *Megaselia* species with any certainty. This process can be extremely time consuming and often involves reading dozens of descriptions for each specimen you are attempting to key. Scanning descriptions day in and day out, dealing with so many specimens and species of *Megaselia*, the authors came to rely upon certain characters (and essentially disregard others) for their identifications. If a specimen matched (or came close to) the key characters of a description, actual specimens were consulted for a definitive diagnosis. It was realized that a streamlined and standardized character set for this group that easily pared down potential matches and heavily utilized visual aides for diagnosis (rather than highly variable verbose descriptions) would facilitate not only identification of known species, but description of new ones as well. If a picture is worth a thousand words, we can remove problems like "light brown" versus "brown", "shiny" versus "glossy", "medium long" versus "medium short" and replace them with a well taken habitus photo. Combine that photo with a wing photo, a drawing of the genitalia, and a photo of a dissected hypandrium, and it's almost like having an actual specimen in front of you. Finish those visual aides off with a table of well defined, objective character states, and the species will practically diagnosis itself.

The task of developing an unfailing system of description for organisms as diverse and numerous as phorids, is a problem opined as impossible by Malloch (Malloch 1912). Although creating a system usable by even the most novice user may, indeed, be impossible, it is the hope of the authors that the task might be conquered at least for those with a basic knowledge of phorid morphology; thus the streamlined description method presented herein. The authors hope that comparable systems might be adopted for similarly challenging taxa in Insecta and beyond, as such standardized descriptions allow rapid assimilation into taxonomic databases and larger scale projects.

## Materials and methods

Specimens were collected from Zurquí de Moravia, San José, Costa Rica in Malaise Traps, preserved in 95% ethanol, and subsequently HMDS dried (Brown 1993) or slide mounted in Berlese’s Fluid (D. J. & D. Henshaw, Waltham Abbey, England) for further study. A subset of specimens were softened in lactic acid and their hypandria were dissected out to be photographed. Specimens were examined using a Leica M205C stereo microscope and photographed on this microscope using a Nikon D600 digital SLR camera. Photograph stacking was done with Helicon Focus software. Specimens are deposited in the Instituto Nacional de Biodiversidad, Costa Rica (INBC) and the Natural History Museum of Los Angeles County, USA (LACM).

### Terms

Standard morphological terminology, as found in the Manual of Central American Diptera (Cumming and Wood 2009) is used. An exception to this would be use of the term “postpedicel” rather than “1^st^ flagellomere”, which is an equally acceptable term that the authors deem a more correct description of this segment (Stuckenberg 1999).

Common terms like “hairs” and “bristles” are used to delineate different types of socketed setae. “Hairs” we use to refer to smaller, non-feathered setae, while “bristles” are much larger, more robust and feathered setae. Although both categories can range in length and thickness, the feathering on a true “bristle” can be readily observed on a standard compound microscope at 40×.

### Characters

The presented character set and states were developed by combining the classic characters outlined by those such as Lundbeck (Lundbeck 1922), Schmitz (Schmitz 1951) and Borgmeier (Borgmeier 1964) with characters that have been shown to be useful more recently, in the authors’ own studies or in descriptions of others working on the group. Additionally, character states for some of the classically utilized characters were refined to remove historical ambiguities and allow them to be used more reliably.

The authors had found certain characters useful to organize large groups of *Megaselia*, some to help differentiate between similar species, some to organize species into clusters for further examination, and some to most simply allow rapid identification when working through vast amounts of material. Some characters that were commonly included in traditional descriptions are simply too subjective or too variable to be used reliably and efficiently, are therefore taxonomically superfluous, and have been omitted from this refined character matrix. The distillation of traditional systems into a workable, clarified set and addition of more recently introduced characters completed the authors’ description and identification system. With a working system in place, author Hartop further refined her character set and states when visiting and working with R. H. L. Disney, who himself uses a shorthand notation of key characters when working with *Megaselia*.

As this system had proven utility in their own studies, the authors decided to formally present a new format for published descriptions of this fauna based on ease of use, and in anticipation of the tremendous number of descriptions forthcoming in this genus. The system of description that is proposed organizes key characters into tables supplemented by visual representations of genitalia and any other salient features, as well as clear habitus, hypandrium and wing photographs. While the authors do not intend the tables themselves to be definitively diagnostic in all cases, the combination of the table and visual aids will be so, and will replace unnecessarily verbose descriptions. This will allow any user to efficiently not only compose new descriptions for the fauna, but to more easily scan vast amounts of existing literature composed in this manner.

In addition to tabulated descriptions, all character states have been coded for use in a character state matrix that will be searchable as an online guide (ie. character "x" has states "a", "b", etc.). This online system (found at www.phorid.net/new-megaselia/home.php) allows users to quickly narrow down species matches using the character matrix, and use the visual aids to finish the identification process. This online key will include built-in tolerance to allow for interspecific variation. All species described using this system by the authors will be uploaded into this system using the coded characters. It is at the discretion of the authors to present the coded states here, but not to utilize them in future published descriptions. They will be used in the construction of the online system, and the authors here present them to allow others to code their own descriptions in the same manner.

Rare *Megaselia* characters are only included in the table if a positive value for such a character state will immediately and definitively place the species into a small group recognizable by that character (such as the lack of wing vein R_2+3_). Any character that is rare but may be subject to ambiguity and therefore potentially generate confusion has been omitted. An example of such a character is the relative lengths of tarsomeres four and five on species such as *M. scutellaris* (Wood). Although for the seasoned *Megaselia* taxonomist the distinction between species like *M. scutellaris*, which has tarsomere five significantly longer than four, may be obvious, for a novice, species where five appears equal or even slightly longer than four may be misleading. On such potentially ambiguous rare characters, the authors opted not to include another required measurement (which would quantify the character and remove said ambiguity but decrease the ease of use of the table) and instead decided that species descriptions containing such rare characters will do so in the remarks section of the table (either in character-specific or general remarks). *Extremely rare* characters (such as the presence of *M. shadeae*’s wing spots) are not included in the table even if definitive, as the presence of such characters is so rare they essentially diagnose the species on their own. Such rare characters will be searchable in the online database with keywords generated from the "remarks" section of the description (see below). This will allow, for example, *Megaselia shadeae* to be found immediately by searching “wing spot”.

Lastly, characters that are definable but not practically usable (such as absolute colors, which would require the use of color palettes accounting for qualities such as hue, tint, shade, and tone while accommodating variation in digital viewing, printing, and specimen lighting), are omitted. Relative coloration (such as halter to scutum) is much less subjective and easily used for coding. It is important to note that relative coloration will occasionally create a discrepancy between a coded character state and what a user observes. Examples of dark species with dark halters that code as “lighter”, because they happen to be lighter than the scutum, do occur. Similarly, light species with light halters that happen to be slightly darker than the scutum and therefore key as “darker” do exist. The authors therefore include the relative coloration of halter to scutum as an easy to use, unambiguous character that agrees with visual perception in most cases. The habitus photographs included with each description will serve as an excellent way to easily compare qualitative characters, including color, for many parts of the specimen.

In order to utilize this description method, specimens should ideally be available in chemically dried, slide mounted, and ethanol preserved (for dissection) states. Habitus photos of chemically dried specimens are essential for conveying the overall coloration, shape, and gestalt of a species while more detailed characters must be observed on slide mounted specimens with a compound microscope. A photograph of the dissected hypandrium can be an essential tool for identification, and this requires additional specimens available for dissection. Therefore, with this system the ideal number of specimens would be a minimum of three. It is the practice of the authors in cases where limited specimens are available to photograph the specimens for habitus images while they are still in ethanol and then slide mount those specimens for detailed observations and measurements. On occasions where only a single specimen is available, this may mean the omission of a separate, dorsoventrally oriented hypandrial photograph.

### Template description

Template for *Megaselia* descriptions (Table 1). Any and all characters may include remarks when described (not included in coded states and thus in a separate column in the tables), and general species remarks may be included at the bottom of the table. The descriptions below are intentionally verbose and thoroughly explanatory to remove potential ambiguity, with the intention that the working table will be usable quickly and easily by those who familiarize themselves with the language of this system. Abbreviations: in text.

Descriptions of characters/states:

**Head**

Supra-antennal (SA) setae ratio: Rather than use the more subjective visual comparisons used classically, the length of the ventral SA are entered as a percentage (given as a decimal to second digit) of the length of the dorsal SA. Therefore, SA that are equal in length will have a value of “1.00”, ventral SA that are half the length of the dorsal SA will have a value of “0.50”, etc.

**Figure 1.**
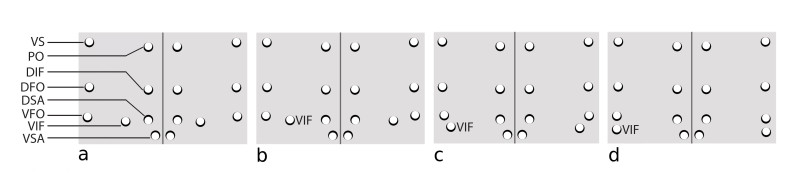
Frontal setation patterns showing "normal" (a & b) and "VFO adjacent" (c & d) arrangements. vs = vertical seta, po = postocellar seta, dif = dorsal interfrontal seta, dfo = dorsal fronto-orbital seta, dsa = dorsal supra-antennal seta, vfo = ventral fronto=orbital seta, vif = ventral interfrontal seta, vsa = ventral supra-antennal seta.

Positioning of ventral interfrontal (VIF) setae: Coded as either ”Normal” orientation (a) or “VFO adjacent” (b). Normal orientation is indicated by the ventral interfrontal (VIF) setae being located either roughly midway between the supra-antennal (SA) setae and the ventral fronto-orbitals (VFO), or closer to the SA (Fig. 1 a & b). If VIF setae are *skewed significantly* closer to (or even directly under) the VFO setae, that is “VFO adjacent” (Fig. 1 c & d). Note, the midrange position includes species that may have the VIF setae slightly closer to the eye margin than the center of the frons, but this is not “VFO adjacent”. “VFO adjacent” is a dramatically skewed position toward the eye margins.

Postpedicel Subcuticular Pit Sensillae (SPS) vesicles: absent (a) or present (b) (Pfeil et al. 1994, Disney 2003).

Palpal setae: “long” (a) if longer than the width of the palpi, “short” (b) if shorter than the width of the palpi.

Labellum: “not spinose” (a) if spinose setulae not present, “sparse” (b) if setulae are scattered and present in numbers fewer than 30 per labellum, “dense” (c) if setulae are densely covering labellum (Disney 1999).

**Thorax**

Anepisternum: An anepisternum without setae is scored as “bare” (a). If setae are present, they can be “hairs only” (b) or they can be “hairs + bristle(s)” (c). If bristles are present, the number and size may be indicated (ie. “hairs + one long bristle”) as a remark, that data will not be coded.

Halter color: halter color is based on the knob of the halter when compared with the color of the scutum. Halteres that are lighter than the scutum are coded as “lighter” (a), halteres that are the same color as the scutum key as "same", and halteres that are darker than the scutum are coded as “darker” (c). It is important to note that in light colored species, halteres may be quite light in color in an absolute sense, but in a relative sense will be scored in the “darker” category, or vice versa. Halter color is best observed on either dried specimens or specimens preserved in ethanol.

Number of notopleural (NP) setae: scored as 2 (a) or 3 (b).

Notopleural (NP) cleft: scored as absent (a) or present (b).

Scutellar setae: scored as “4 =” (a) if anterior and posterior are the same size, “4 /=” [note "/=" is a more computer-friendly version of the mathematical "not equal" symbol], (b) if the anterior and posterior pairs are not the same but the smaller pair are longer than the posterior small setulae on the scutum, and “2+2” (c) if the smaller pair are minute and equal in length to the posterior small setulae on the scutum.

**Legs**

Foretarsus (ts1) palisade: indicate the segment numbers of the foretarsus on which a setal palisade is present (i.e. Tarsomeres 1–4, etc.).

Midtibial (t2) palisade: indicate portion of tibia on which a setal palisade is present (0.50, 0.75, etc.).

Bifurcated spines in comb of hind tibia (t3): “absent” (a), or “present” (b).

Hind tibial (t3) setulae: a single, posterodorsal, row of setulae on the hind tibia is “PD only” (a); if a second row is present on the anteroventral side of the tibial palisade, that is “PD + AD” (b).

Hind femoral (f3) basal setae: length of basal setae on hind femur indicated relative to setae on the anteroventral surface of the hind femur as “B<AV” (a), “B=AV” (b) or “B>AV” (c).

Hind femoral (f3) basal setae differentiation: “absent” (a) or “present” (b), description in remarks. Examples of differentiation would be setae that are clearly thickened, curved, found in unusual configurations, etc.

**Wing**

Wing length: given in mm to second decimal, measured as per Schmitz (fig. 45 in Schmitz 1951); from large basal bristle on basicosta to wing tip, taking length parallel to the costa.

Subcosta (Sc): given as “complete” (a) when it reaches vein R_1_ or “incomplete” (b) when it fades out before reaching R_1_.

Hair at base of vein R: either “absent” (a), “minute” (b) if shorter than the width of the R vein, “short” (c) if longer than the width of the vein but shorter than 2× the width of the vein, or “long” (d) if longer than 2× the width of the vein.

R_2+3_ vein: either “present” (a) or “absent” (b).

Costal index (CI): given as a decimal to second digit, found by dividing the wing length by the length of the costal vein (fig. 45 in Schmitz 1951). As this is a relative, not absolute, measurement it can most easily be taken on a printed photograph of the wing.

Costal ratios: given as “C1:C2:C3” with C3 at a value of 1 and others to second decimal (fig. 44 in Schmitz 1951). As with the CI, these are a relative measurement and are most easily taken using a printed photograph of the wing.

Costal setae length: measurement in mm of the length of the longest costal setae present in section 3 of the costa.

Number of alular setae: given as a number, coded as “1” (a), “2” (b), “3” (c), “4+” (d) due to observed variation in number of alular setae on species with more than four present.

Alular setae length: given in mm, longest seta present is measured.

Wing color: either “lightly infuscated/clear” (a) or “strongly infuscated” (b); the authors understand that this character may include some ambiguity, so therefore it must be clarified that most wings are state "a", and only wings that are distinctly infuscated should be coded as the second character state. Wings are best viewed on dried specimens (or in a clear mounting medium, ie. *not* Canada Balsam) against a white background to observe this character.

**Genitalia**

Anal tube (AT) length relative to length of dorsal face of epandrium (E): length of the anal tube (cerci + hypoproct) is given relative to the dorsal face of the epandrium as “AT<E” (a), “AT=E“ (b), “AT>E” (c). This comparison is most easily done on a slide mounted specimen, where the epandrium can be seen even if it is partially covered by the tergites and must be viewed through them.

Epandrial (E) Setation: “hairs only” (a) or “hairs + bristle(s)” (b).

Relative lengths of posterior setation: indicate the relative lengths (not thickness) of setae found on Tergite 6 (T6), Epandrium (E), Cerci (C) and Hypoproct (H) with operators < [less than], = [equal], or ~ [subequal: approximately equal] (ex. T6<C=H<E).

## Taxon treatments

### Megaselia shadeae

Hartop sp. n.

urn:lsid:zoobank.org:act:7ADB2743-C5BB-4515-A2E6-A26055B8CA61

#### Materials

- **Type status:** Holotype. **Occurrence:** catalogNumber: 322007; individualCount: 1; sex: male; lifeStage: adult; **Taxon:** scientificName: *Megaselia shadeae* Hartop 2014; **Location:** country: Costa Rica; stateProvince: San Jose; locality: Zurqui de Moravia; verbatimElevation: 1600 m; verbatimCoordinates: 10.05°N, 84.01°W; decimalLatitude: 10.05; decimalLongitude: -84.01; georeferenceProtocol: GPS; **Identification:** identifiedBy: Brian Brown; dateIdentified: 2014; **Event:** samplingProtocol: Malaise trap #1; eventDate: 2013-06-14/21; **Record Level:** institutionCode: LACM; collectionCode: ENT; basisOfRecord: PreservedSpecimen
- **Type status:** Paratype. **Occurrence:** catalogNumber: 322008; individualCount: 1; sex: male; lifeStage: adult; **Taxon:** scientificName: *Megaselia shadeae* Hartop 2014; **Location:** country: Costa Rica; stateProvince: San Jose; locality: Zurqui de Moravia; verbatimElevation: 1600 m; verbatimCoordinates: 10.05°N, 84.01°W; decimalLatitude: 10.05; decimalLongitude: -84.01; georeferenceProtocol: GPS; **Identification:** identifiedBy: Brian Brown; dateIdentified: 2014; **Event:** samplingProtocol: Malaise trap #1; eventDate: 2013-06-14/21; **Record Level:** institutionCode: LACM; collectionCode: ENT; basisOfRecord: PreservedSpecimen
- **Type status:** Paratype. **Occurrence:** catalogNumber: 275333; individualCount: 1; sex: male; lifeStage: adult; **Taxon:** scientificName: *Megaselia shadeae* Hartop 2014; **Location:** country: Costa Rica; stateProvince: San Jose; locality: Zurqui de Moravia; verbatimElevation: 1600 m; verbatimCoordinates: 10.05°N, 84.01°W; decimalLatitude: 10.05; decimalLongitude: -84.01; georeferenceProtocol: GPS; **Identification:** identifiedBy: Brian Brown; dateIdentified: 2014; **Event:** samplingProtocol: Malaise trap #1; eventDate: 2012-09-12/18; **Record Level:** institutionCode: LACM; collectionCode: ENT; basisOfRecord: PreservedSpecimen
- **Type status:** Paratype. **Occurrence:** catalogNumber: 275324; individualCount: 1; sex: male; lifeStage: adult; **Taxon:** scientificName: *Megaselia shadeae* Hartop 2014; **Location:** country: Costa Rica; stateProvince: San Jose; locality: Zurqui de Moravia; verbatimElevation: 1600 m; verbatimCoordinates: 10.05°N, 84.01°W; decimalLatitude: 10.05; decimalLongitude: -84.01; georeferenceProtocol: GPS; **Identification:** identifiedBy: Brian Brown; dateIdentified: 2014; **Event:** samplingProtocol: Malaise trap #1; eventDate: 2012-09-12/19; **Record Level:** institutionCode: LACM; collectionCode: ENT; basisOfRecord: PreservedSpecimen
- **Type status:** Paratype. **Occurrence:** catalogNumber: 326547; individualCount: 1; sex: male; lifeStage: adult; **Taxon:** scientificName: *Megaselia shadeae* Hartop 2014; **Location:** country: Costa Rica; stateProvince: San Jose; locality: Zurqui de Moravia; verbatimElevation: 1600 m; verbatimCoordinates: 10.05°N, 84.01°W; decimalLatitude: 10.05; decimalLongitude: -84.01; georeferenceProtocol: GPS; **Identification:** identifiedBy: Brian Brown; dateIdentified: 2014; **Event:** samplingProtocol: Malaise trap #1; eventDate: 2012-09-12/20; **Record Level:** institutionCode: LACM; collectionCode: ENT; basisOfRecord: PreservedSpecimen

#### Description

**Figure 2.**
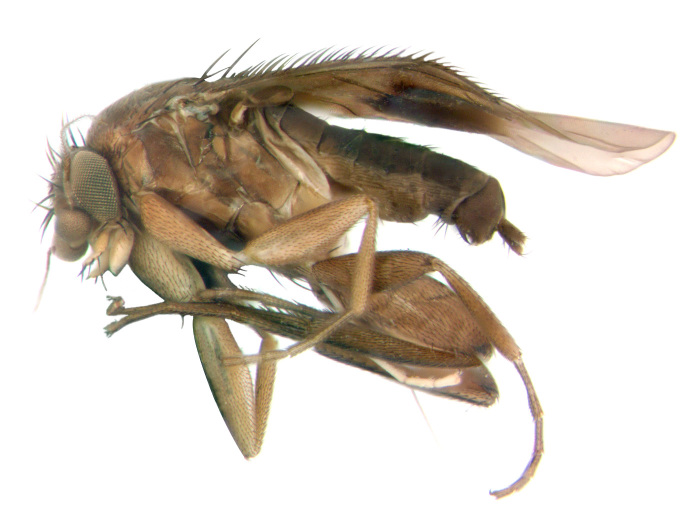
*Megaselia shadeae* new species, male, left lateral.

**Figure 3.**
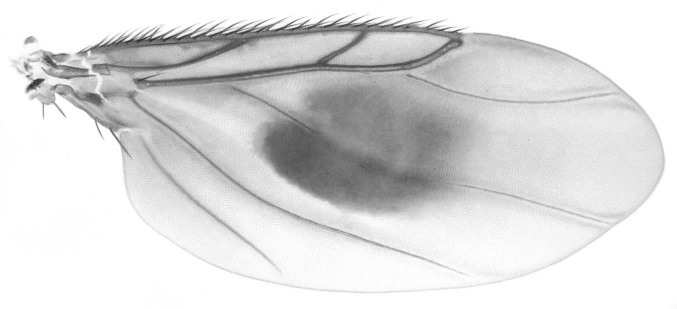
*Megaselia shadeae* new species, male, right wing, dorsal.

**Figure 4.**
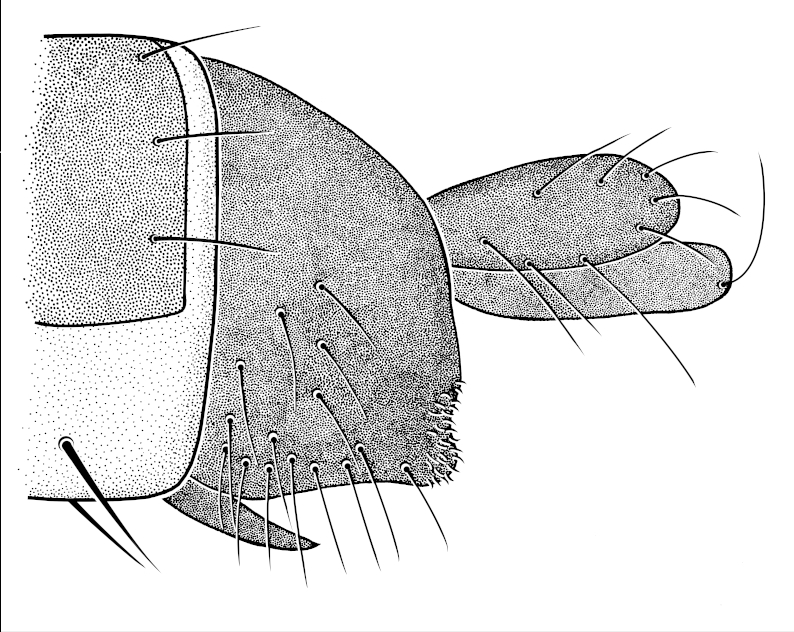
*Megaselia shadeae* new species, male genitalia and tip of abdomen, left lateral.

**Figure 5.**
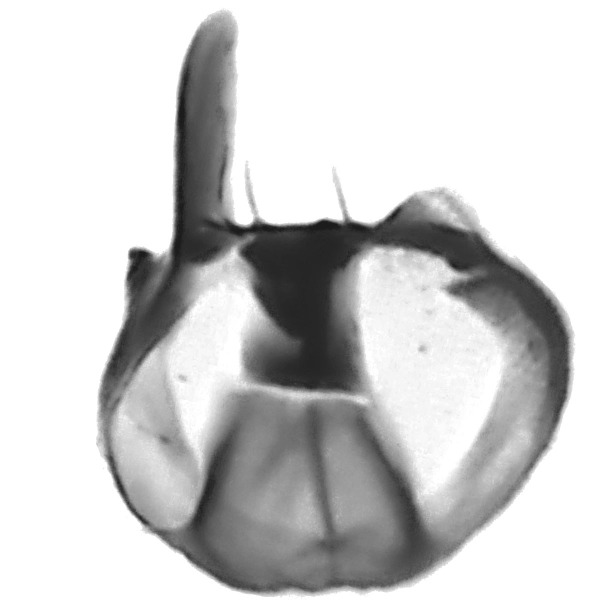
*Megaselia shadeae* hypandrium (ventral).

See Table 2, Figs 2, 3, 4, 5.

#### Diagnosis

Wing with darkly-pigmented central swelling in center. Fork formed by wing veins R_2+3 _and R_4+5_ greatly enlarged. The central wing spot makes this different from all other described Neotropical species with the single exception of *M. dicksoni* (Wakeford and Disney 1994), from which it differs in having a bubbled and pigmented, rather than a scaled, wing spot.

#### Etymology

Named for E. A. Hartop's niece, Shade Zehendner.

#### Distribution

Known from a single site in Costa Rica.

#### Biology

Unknown.

#### Taxon discussion

A primary key to Neotropical species of *Megaselia* was given by Borgmeier (1962), who supplemented his original key with two additional keys to Neotropical species (Borgmeier 1969a, Borgmeier 1971), and a key to Dominican species (Borgmeier 1969b). In Borgmeier (1962), this species keys to couplet 62 of the group VII key where it differs immediately from both *M. notipennis* and *M. phoebe* by the presence of a wing spot.

Neotropical species of *Megaselia* described subsequent to Borgmeier’s keys are given by Boesi et al. (2006), Brown and Horan (2011), Kung and Brown (2004), Disney (1982), Disney (1989), Disney (1995), Disney and Berghoff (2007), Disney and Rettenmeyer (2007), Disney and Rettenmeyer (2010), Disney and Sakai (2001), Disney and Sinclair (2008), Disney and Weinmann (1998), Downie et al. (1995), Gonzalez et al. (2002), Wakeford and Disney (1994), Weinmann and Disney (1997), and Woolf (1998). This species is easily distinguished from all of these described species except *M. dicksoni* (Wakeford and Disney 1994) by the presence of a central wing spot. In practice, *M. shadeae* is differentiated easily from *M. dicksoni* by the composition of the characteristic wing spot. In *M. shadeae*, the wing spot is pigmented wing membrane, whereas in *M. dicksoni* the wing spot is composed of a patch of pigmented scales.

Three genera that have been synonymized (or partially synonymized) with *Megaselia* that contain Neotropical fauna are *Pericyclocera* Schmitz, *Paraphiochaeta* Malloch, and *Plastophora* Brues. The species herein described is easily distinguished from species once classified in these genera by presence of the wing spot.

## Discussion

The presence of such a remarkable and distinct undescribed species of Neotropical *Megaselia* is indicative of the paucity of taxonomic resources currently available for this fauna. Despite the frequent occurrence and ease of identification of this species, this fly was, until now, undescribed. The immense amount of taxonomic work needed for this genus is apparent — the hundreds of other species found alongside *M. shadeae* in the ZADBI project await description. Unfortunately, and this is the case for this fauna worldwide, most of the world’s *Megaselia* are poorly known. It is the hope of the authors that the streamlined presentation of species data presented here will help stimulate rapid and abundant descriptions of unknown fauna as well as facilitating the identification of unknowns.

## Supplementary Material

XML Treatment for Megaselia shadeae

## Figures and Tables

**Table 1.** Template for *Megaselia* descriptions.

***Species name*** Fig X (habitus).		
**Head**		**Remarks**
SA ratio	decimal to second digit	
VIF position	normal or VFO adjacent	
SPS vesicles	absent or present	
Palpal setae length	long or short	
Labellum spinosity	not, sparse, or spinose	
**Thorax**		
Anepisternum	bare, hairs only, or hairs + bristles	
Relative halter color	lighter, same, or darker	
# NP setae	number	
NP cleft	absent or present	
Scutellar setae	4 =, 4 /=, or 2+2	
**Leg**		
ts1 palisade	number of tarsomeres	
t2 palisade	portion of tibia present (decimal to second digit)	
t3 comb bifurcate	absent or present	
t3 setulae	PD or PD+AD	
f3 basal setae	B <, =, or > AV	
f3 basal setae differentiation	absent or present	
**Wing**	Fig X	
Wing Length (mm)	decimal to second digit	
Subcosta	complete or incomplete	
Hair at base of R	absent, minute, short or long	
R_2+3_	present or absent	
Costal index	decimal to second digit	
Costal ratios	C1:C2:C3 (C3=1)	
Costal setae length (mm)	decimal to second digit	
Number alular setae	number	
Alular setae length (mm)	decimal to second digit	
Wing color	lightly infuscated/clear or strongly infuscated	
**Genitalia**	Fig X, X (genitalia, hypandrium)	
AT length	AT <, =, or > E	
E setation	hairs only or hairs + bristles	
Relative posterior setation	T6, E, C, H in ascending order using (<, =, ~)	
**General Remarks**		
any information unique to species		

**Table 2.** Description of *Megaselia shadeae* sp. n.

***M. shadeae*** Fig. 2		
**Head**		**Remarks**
SA ratio	1	
VIF position	VFO adjacent	
SPS vesicles	absent	
Palpal setae length	long	
Labellum spinosity	spinose	
**Thorax**		
Anepisternum	bare	
Relative halter color	same	
# NP setae	2	
NP cleft	absent	
Scutellar setae	2+2	
**Leg**		
ts1 palisade	1-4	
t2 palisade	0.67	
t3 comb bifurcate	absent	
t3 setulae	PD	
f3 basal setae	B<AV	
f3 basal setae differentiation	absent	
**Wing**	Fig. 3	
Wing Length (mm)	1.56	
Subcosta	incomplete	
Hair at base of R	long	
R_2+3_	present	large fork
Costal index	0.65	
Costal ratios	1.25:1.00:1	
Costal setae length (mm)	0.09	
Number alular setae	3	
Alular setae length (mm)	0.15	
Wing color	strongly infuscated	
**Genitalia**	Figs 4, 5	
AT length	AT>E	
E setation	hairs only	
Relative posterior setation	T6~E~H<C	
**General Remarks**		
wing with central, bubbled, pigmented spot		
